# Transautophagy: Research and Translation of Autophagy Knowledge

**DOI:** 10.1155/2018/7504165

**Published:** 2018-05-16

**Authors:** Maria C. Albertini, Tassula Proikas-Cezanne, Nikolai Engedal, Eva Žerovnik, Jon D. Lane

**Affiliations:** ^1^University of Urbino “Carlo Bo”, Urbino, Italy; ^2^Eberhard Karls University of Tübingen, Tübingen, Germany; ^3^International Max Planck Research School “From Molecules to Organisms”, Tübingen, Germany; ^4^University of Oslo, Oslo, Norway; ^5^Jožef Stefan Institute, Ljubljana, Slovenia; ^6^University of Bristol, Bristol, UK

There is an urgent need to understand the process of autophagy in health and disease. Increasingly, human age-related diseases, such as cancer and neurodegeneration, as well as the great variety of metabolic disorders are found to correlate with significant alterations in both autophagy activity and capacity. However, implementing novel therapeutic treatment opportunities for combatting age-related human pathologies by targeting autophagy is unsatisfactorily slow. One of the reasons for this is that autophagy methods that can precisely monitor autophagy activity and capacity *in vivo* are currently nonexistent. Moreover, molecular details with regard to the complex regulation of autophagy in health and disease are still poorly understood. As a consequence, applying personalized medicine for targeting autophagy in patients suffering from age-related human diseases is still an unfulfilled aim.

In recognition of the requirement for developing next-generation autophagy knowledge and methodologies, the COST (European Cooperation in Science and Technology) Action (CA15138) “Transautophagy” (http://cost-transautophagy.eu/), a European network for multidisciplinary research on autophagy, has been successfully established by Caty Cases Louzao, Spain, and Patrice Codogno, France, in 2016. More than 200 autophagy researchers from over 28 European and neighboring countries aim to significantly advance autophagy research, concentrating on basic research on the autophagy molecular machinery (working group 1), strategies for autophagy analyses and modulation (working group 2), autophagy applications to crop and energy production (working group 3), biomedical research (working group 4), and biomedical translation and clinical trials (working group 5).

In this special issue, important further information on molecular mechanisms regulating autophagy is provided. These mechanisms highlight the relationship existing between cellular ROS levels and the process of autophagy. M. Pajares et al. reviewed publications related to the role of redox signaling in autophagy regulation with a special interest on ageing-associated changes and Alzheimer's disease. J. Jin et al. used primary acute myeloid leukemia (AML) patient samples and human AML cell lines to investigate the requirement for autophagy in AML differentiation. They suggested that granulocytic AML differentiation relies on noncanonical autophagy pathways and that restoring autophagic activity may be beneficial in therapies that aim at stimulating differentiation. N. Engedal et al. described novel aspects of oxidative stress-modulated miRNAs and elegantly demonstrated how an in silico approach can be employed to formulate hypotheses-driven research on the interrelation between miRNA-based gene regulation, oxidative stress signaling pathways, and autophagy. T. Zuleger et al. investigated the role of the serum- and glucocorticoid-induced protein kinase 1 (SGK1) in the control of autophagy. Their data strongly support the idea that SGK1 inhibits the process of autophagy. SGK1 seems to act upstream of ULK1 in regulating autophagy, and the authors provide a model whereby SGK1 regulates autophagy by contributing to the control of ULK1 gene expression. R. Mancinelli et al. reviewed the multifaceted roles of GSK-3 in cancer and autophagy-related diseases, in the context of the role of GSK-3 in several signaling pathways controlling a great variety of different key cellular functions.

Polyphenols, and other antioxidant molecules, have been considered as compounds that modulate the process of autophagy. M. Holczer et al. investigated the role of epigallocatechin-3-gallate (EGCG), the major polyphenol of green tea, in promoting autophagy-dependent survival. Their findings, obtained using HEK293T cells *in vitro*, revealed that EGCG treatment induced cytoprotective autophagy and increased cell viability by downregulating mTOR and upregulating AMPK signaling. S. Hasanbašić et al. showed that a set of polyphenolic antioxidants, in particular curcumin, can combat the process of protein aggregation *in vitro*. However, some antioxidants, such as vitamin C and NAC may have opposing effects at higher concentrations. The authors suggest that the level of protein aggregation may act as a sensor in order to ultimately prevent further cellular damage. C. Miceli et al. explored the effects of OA (Oleuropein aglycone, the main polyphenol found in olive oil) in cardiomyocytes by overexpression of monoamine oxidase-A (MAO-A). The authors observed that OA treatment counteracted the cytotoxic effects of MAO-A by restoring the MAO-A-induced defect in autophagic flux, most probably via activation and nuclear translocation of TFEB (transcription factor EB).

Finally, further studies focused on the role of oxidative stress and autophagy in different pathologies and new therapeutic strategies. M. Marinkovic et al. summarized the current knowledge of the interplay between autophagy regulation and five of the most life-threatening and prevalent malignancies (pancreatic, breast, hepatocellular, colorectal, and lung cancer). In addition, the authors present an overview of the recent advances in therapeutic strategies involving autophagy modulators in cancer therapy. A. Van Erp et al. reviewed knowledge on the crosstalk regulation between oxidative stress and autophagy in the context of transplantation medicine. C. Fang et al. discussed the implication of ROS and autophagy in both the onset and development of neurological disorders. The authors highlighted the interplay between ROS and autophagy in establishing a determinant role in the modulation of neuronal homeostasis, by an as yet unexplored mechanism, important in cerebral ischemia, AD (Alzheimer's disease), and PD (Parkinson's disease).

In summary, this special issue provides several new aspects in autophagy research with emphasis on translation of knowledge to applications, critically including the topic of autophagy regulation upon oxidative stress, aging, age-related diseases, and beyond (Figure 1).

## Figures and Tables

**Figure 1:**
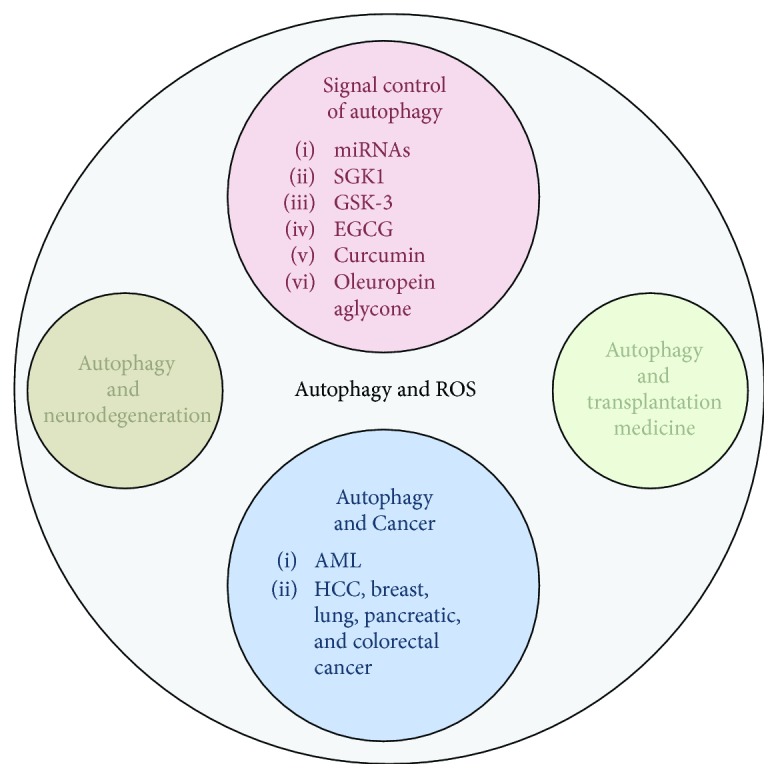
Summary of topics that either have been discussed in review articles or have been addressed in original studies in this special issue.

